# FCN3 inhibits the progression of hepatocellular carcinoma by suppressing SBDS-mediated blockade of the p53 pathway

**DOI:** 10.7150/ijbs.69784

**Published:** 2023-01-01

**Authors:** Dong Ma, Pengpeng Liu, Junjun Wen, Yang Gu, Zhangshuo Yang, Jianwei Lan, Haining Fan, Zhisu Liu, Deliang Guo

**Affiliations:** 1 Department of Hepatobiliary and Pancreatic Surgery, Zhongnan Hospital of Wuhan University, Wuhan, 430071, P.R. China.; 2 Department of Hepatobiliary and Pancreatic Surgery, Affiliated Hospital of Qinghai University, Xining, 810000, P.R. China.; 3 Department of Hepatobiliary Surgery, Tianjin Medical University General Hospital, Tianjin, 300052, P.R. China.; 4 Department of Hepatobiliary and Pancreas, The First People's Hospital of Jingmen, Jingmen, 448000, P.R. China.

**Keywords:** Hepatocellular carcinoma, FCN3, SBDS, p53 signaling pathway, Ribosomal stress

## Abstract

Hepatocellular carcinoma (HCC) is the third-leading cause of cancer deaths globally. Although considerable progress has been made in the treatment, clinical outcomes of HCC patients are still poor. Therefore, it is necessary to find novel prognostic factors upon which prevention and treatment strategies can be formulated. Ficolin-3 (FCN3) protein is a member of the human ficolin family. It activates complement through pathways associated with mannose-binding lectin-associated serine proteases. Herein, we identified that FCN3 was downregulated in HCC tissues and decreased FCN3 expression was closely related to poor prognosis. Overexpression of FCN3 induced apoptosis and inhibited cell proliferation via the p53 signaling pathway. Mechanistically, FCN3 modulated the nuclear translocation of eukaryotic initiation factor 6 (EIF6) by binding ribosome maturation factor (SBDS), which induced ribosomal stress and activation of the p53 pathway. In addition, Y-Box Binding Protein 1 (YBX1) involved in the transcription and translation level regulation of FCN3 to SBDS. Besides, a negative feedback loop in the downstream of FCN3 involving p53, YBX1 and SBDS was identified.

## 1. Introduction

Primary liver cancer is the third-leading cause of cancer deaths globally and hepatocellular carcinoma (HCC) is the most common histological form of primary liver cancer 1. There are various risk factors related to the occurrence and development of HCC. In the highest incidence Asia and Africa region, it is evidently associated with Hepatitis B and C virus 2. Although considerable progress has been made in the treatment, including surgery, transcatheter arterial chemoembolization (TACE), molecular targeted drugs and immunotherapy, clinical outcomes of HCC patients are still poor 3, 4. Therefore, it is necessary to find novel prognostic factors and potential molecular mechanisms to improve the prevention and treatment of HCC.

Ficolin-3 (also called H-ficolin) (FCN3), a protein encoded by FCN3 gene, is mainly expressed in the lung and liver in human tissues. It can activate complement through pathways associated with mannose-binding lectin-associated serine proteases (MASPs) 5, 6. FCN3 has been reported to be downregulated in lung adenocarcinoma, esophageal carcinoma, and serum of HCC patients 7-11. Kim Ahreum. *et al.* 12 found that FCN3 inhibited tumor progression in lung squamous cell carcinoma by influencing the tumor immune microenvironment. The serum expression level of FCN3 was correlated with the progression of Hepatitis C virus (HCV) cirrhosis to HCC, and was also a biomarker for evaluating the prognosis of HCC after radiofrequency ablation (RFA) and TACE 13-15. However, the expression pattern of FCN3 in HCC tissues and its contribution to tumor progression remains unclear.

In this study, we found that low expression of FCN3 was associated with poor prognosis in HCC patients. FCN3 promoted apoptosis and inhibited cell proliferation of HCC cells. Mechanistically, by binding to SBDS, FCN3 inhibited the entry of eukaryotic initiation factor 6 (EIF6) into the nucleus, which in turn induced ribosomal stress and activated the p53 signaling pathway. Moreover, FCN3 further activated the p53 signaling pathway by suppressing SBDS expression through downregulation of the transcription factor Y-Box Binding Protein 1 (YBX1). Further, p53 inhibited the expression of YBX1, resulting in the formation of a p53/YBX1/SBDS negative feedback loop in the downstream of FCN3.

## 2. Materials and Methods

### 2.1. Tissue samples and clinical data

In this study, paired tumor and para-tumor tissue samples in 202 patients with HCC were collected from Zhongnan Hospital of Wuhan University. All tissue samples were confirmed with HCC through histopathologic examination and stored at -80 °C in RNAlater solution (Invitrogen, USA) until further assays. Moreover, clinical data of patients were collected from the electronic medical records in Zhongnan Hospital of Wuhan University. Informed consent was obtained from all patients in this study. Ethical approval was granted by the Ethics Committee of Zhongnan Hospital of Wuhan University.

### 2.2. Cell culture

Human HCC cell lines including HCCLM3, SK-Hep1, Huh7, SMMC7721, Hep3B, HepG2, and normal liver cell line L02 were purchased from the Cell Bank of Type Culture Collection of Chinese Academy of Sciences (Shanghai, China). All cells were cultured in Dulbecco's Modified Eagles Medium (DMEM) (Gibco, USA) supplemented with 10% fetal bovine serum (FBS) (Gibco, USA) and 1% penicillin-streptomycin (Gibco, USA). Cell cultures were maintained in a humidified incubator at 37 °C with 5% CO_2_.

### 2.3. Total RNA extraction and quantitative real-time PCR

Total RNA were extracted from tissue samples and cells using the Trizol reagent (Invitrogen, USA). RNA concentration was determined by absorbance at 260nm, and RNA purity was assessed based on absorbance at 260/280 nm with NanoDrop2000 (Thermo Scientific, USA). Reverse transcription was performed using HiScript II Q RT SuperMix (Vazyme, China) to prepare cDNA. Then, quantitative real-time PCR was performed using 2× SYBR qPCR Master Mix (Vazyme, China) for all samples in triplicate in the CFX96 Real-Time PCR System (Bio-rad, USA). Human GAPDH gene was used as the control to normalize the mRNA expression of target genes using 2^-ΔΔCT^ method. Primer sequences involved in this study were shown in Supplementary file 1: Table S2.

### 2.4. Plasmid construction and cell transfections

Full-length FCN3 with a C-terminal 6× His tag, SBDS with a C-terminal Myc tag and YBX1 were cloned into pcDNA3.1(+), respectively. For the FRET assay, full-length FCN3 and SBDS were cloned separately into pAcGFP1 and pDsRedMonomer. SiRNAs targeting human FCN3 and YBX1 (siFCN3#1; siFCN3#2; siYBX1#1; siYBX1#2) and control siRNA (siControl) were purchased from Tsingke Biotechnology (Wuhan, China). Lipofectamine 3000 Reagent and Lipofectamine 2000 Reagent (Invitrogen) were used separately for plasmid and siRNAs transfection according to the manufacturer's instructions.

### 2.5. Immunofluorescence (IF) and immunohistochemistry (IHC)

For IF assay, transfected or untransfected cells were grown on 14mm coverslips and fixed with 4% paraformaldehyde for 15 min at room temperature. Coverslips were permeabilized using 0.5% Triton X-100, blocked in 5% bovine serum albumin (BSA). Next, coverslips were incubated with primary antibodies followed by fluorescein-conjugated secondary antibody. Nuclear staining was performed with DAPI for 15 min. Finally, cells were washed 3 times with PBS gently and imaged with a confocal laser scanning microscopy (Leica sp8, Germany).

For IHC assay, the tissue sections were deparaffinized and rehydrated followed by antigen retrieval and inactivation of endogenous peroxidase in 3% H2O2. After blocking with 5% BSA, sections were incubated with primary antibodies and corresponding secondary antibodies, developed with DAB, stained with hematoxylin. Last, slides were sealed with using neutral resins and scanned using a ScanScope AT Turbo slide scanner (Leica, Aperio VERSA 8).

### 2.6. Western blot assay

Total proteins of tissue samples and cells were extracted by RIPA lysis buffer with 1mM PMSF (Beyotime, China). After quantified by BCA assay (Beyotime, China), proteins were separated using 10% SDS-PAGE gel and transferred to PVDF membrane. Membranes were blocked in 5% BSA in TBST for 1h at room temperature, then incubated with primary antibodies overnight at 4 °C. Subsequently, Membranes were incubated with HRP-conjugated secondary antibodies for 1h at room temperature, and developed using ECL Western Blot Substrate (Bio-rad, USA). Details of primary antibodies were listed in Supplementary file 1: Table S3.

### 2.7. Flow cytometry analysis

After 24h transfection, cells were washed with PBS pre-cooling and harvested to stain using an Annexin V-FITC/PI apoptosis kit (Multi Sciences). The apoptosis rate of cells was analyzed by flow cytometry (Beckman Coulter, USA).

### 2.8. Colony formation assay

Cells transfected as described above were used for colony formation assay. Cells were seeded into 6-well plates with a starting density of 1000-2000 cells per well. Colonies were fixed with 4% paraformaldehyde after 2 weeks and stained with crystal violet. Numbers of colonies were counted using ImageJ software (http://imagej.nih.gov/ij/).

### 2.9. 5-Ethynyl-2′-deoxyuridine (EdU) incorporation assay

EdU is the modified analog of thymidine and gets incorporated into newly synthesized DNA during cell proliferation. EdU Cell Proliferation Kit with Alexa Fluor 594 (Beyotime, China) was used to evaluate proliferation capacity of transfected cells as well as tissue sections of xenograft tumors in nude mice. The images were acquired by a fluorescent microscope (Olympus, Japan). Proportion of EdU positive cells was presented as the ratio of EdU-positive cells (red cells) to total Hoechst-positive cells (blue cells).

### 2.10. Coimmunoprecipitation (Co-IP) and mass spectrometry (MS)

HEK-293T cells were co-transfected with His-FCN3 and cMyc-SBDS to perform exogenous Co-IP assay. Cell lysates were prepared using IP buffer (20mM Tris-HCl, pH 7.4; 1mM EDTA, pH 8.0; 150mM NaCl; 1% NP-40) containing 1% Phosphatase inhibitor cocktail (Meilunbio, China) for 30 min at 4 °C. After centrifugation at 12000g for 10 min at 4 °C, the supernatant was collected and incubated with 20ul magnetic beads and 2-3ul antibodies (His-tag, Myc-tag) overnight at 4 °C. Next day, the supernatant was removed using a magnetic stand, the beads bound specific antibodies and target proteins were resuspended in 1× loading buffer and denatured for 10mins at 95 °C. Finally, the samples were centrifuged at 12000g for 1-2 min at 4 °C and the supernatant stored at -80 °C prior to immunoblot. HCCLM3 and SK-Hep1 cells transfected with His-FCN3 were performed to endogenous Co-IP assay using SBDS antibody or IgG, and the followed steps were the same as the exogenous Co-IP assay. HCCLM3 cells were transfected His-FCN3 and performed Co-IP assay as described above. The eluted protein samples from beads were separated on SDS-PAGE, and subjected to silver staining and mass spectrometry analysis. The score of proteins in MS results was obtained using ProteinPilot SoftwareTM V4.0.8085 (Applied Biosystems, Foster City, CA) as described by Yasir, M. *et al.* 16.

### 2.11. Fluorescence resonance energy transfer (FRET) assay

HCCLM3 cells co-transfected with pAcGFP1 and pDsRedMonomer were plated in confocal dish with glass-bottom (Biosharp) as the control group, while AcGFP1-FCN3 and DsRedMonomer-SBDS were used in the experimental group. AcGFP1 and DsRedMonomer were excited separately at 488nm and 556nm. FRET was measured by a confocal laser scanning microscopy (Leica sp8, Germany) according to previous method 17.

### 2.12. Nucleocytoplasmic separation assay

Cells nuclear and cytoplasmic proteins from HCCLM3 and SK-Hep1 cells transfected FCN3 or vector were extracted using a Nuclear and Cytoplasmic Protein Extraction Kit (Beyotime, China) according to the manufacturer's instructions for Western blot.

### 2.13. Ribosome profiling

HCCLM3 cells transfected with vector or FCN3 were harvested after 48h. Cells were lysed with lysis buffer (15mM Tris-HCl, pH 7.4; 15mM MgCl_2_; 300mM NaCl; 1% NP-40; 40U/ul RNase and protease inhibitors) on ice for 30 min and centrifuged at 10000g for 10 min at 4 °C. The supernatants containing ribosomes were loaded into the 10-50% sucrose gradient. After centrifugation for 4h at 32000 rpm at 4 °C in a SW41 rotor (Beckman Coulter, USA), gradients were analyzed using a NanoDrop2000 (Thermo Scientific, USA) and absorbance was measured at 260nm to obtain the ribosome profile as described by Aquino, G. *et al*. 18.

### 2.14. Dual luciferase reporter activity assay

The wild-type or mutant promoter of the SBDS was cloned into luciferase reporter vectors, and co-transfected with pcDNA3.1-YBX1 into HEK-293T cells. Then dual luciferase reporter activity assay was performed using a Dual luciferase Reporter Assay Kit (Vazyme Biotech, China), luciferase activity was measured and normalized to renilla luciferase activity.

### 2.15. Chromatin immunoprecipitation (ChIP) assay

ChIP assay was performed to detect the YBX1 protein binds to the SBDS promoter in living cells using a Chromatin Immunoprecipitation (ChIP) Assay Kit (Millipore, USA) according to the manufacturer's instructions. DNA fragments were released from antibody-protein-DNA complex, and purified DNA was analyzed by qPCR with primers in Supplementary Table 1.

### 2.16. Tumorigenesis assay

Male BALB/C nude mice were supplied by the Experimental Animal Central of Zhongnan Hospital of Wuhan University. The protocol conformed to the requirements of experimental animal welfare and ethics and was approved by the Experimental Animal Welfare Ethics Committee, Zhongnan Hospital of Wuhan University. Mice were randomly divided into an experimental or control group (5 mice per group). HCCLM3 cells were transfected FCN3 or vector, and 150ul cell suspension (5×10^6^ cells) in DMEM without FBS was injected into the right armpit of mice. For assessment of cell proliferation *in vivo*, EdU (50 mg/kg of body weight) was intraperitoneally injected to mice 4 h prior to collection of subcutaneous xenografts. The weight and volume of subcutaneous xenografts were measured, and then they were fixed in 4% paraformaldehyde, embedded in paraffin and sectioned. Subsequently, EdU assay of sections was performed as described above.

### 2.17. Bioinformatics analysis

The Cancer Genome Atlas (TCGA) database was used to analyze the expression of FCN3 in HCC and single-gene gene set enrichment analysis (GSEA) (http://www.broadinstitute.org/gsea). The analysis and visualization of the expression pattern of FCN3 in multiple cohorts from the Oncomine database (http://www.oncomine.org). The “limma” package was used to analyze the result of transcriptome sequencing in R software 19. DAVID 6.7 was used for Kyoto Encyclopedia of Genes and Genomes (KEGG) pathway enrichment analysis of all differentially expressed genes (DEGs). CIS-BP Database (http://cisbp.ccbr.utoronto.ca/index.php), KnockTF (http://www.licpathway.net/KnockTF/index.html) and GRNdb (http://grndb.com/) were used to filter the potential transcription factors of SBDS. The p53-associated data sets were obtained from GEO database (http://www.ncbi.nlm.nih.gov/geo/).

### 2.18. Statistical analysis

All data in this study were presented as the means ± standard deviation (SD). Statistical analysis was performed with Student's t test for two groups and ANOVA for multiple groups using GraphPad Prism 8.0 (GraphPad Software, USA). The χ2 test was used to analyze the relationship between FCN3 expression and various clinicopathological characteristics in HCC patients. Survival curves were plotted by the Kaplan-Meier method and compared using the log-rank test. Multivariate Cox regression analysis was conducted to evaluate the independent prognostic factors. And P value < 0.05 was considered as statistically significant.

## 3. Results

### 3.1. FCN3 was downregulated in HCC and decreased FCN3 expression was associated with poor patient prognosis

**Figure 1:**
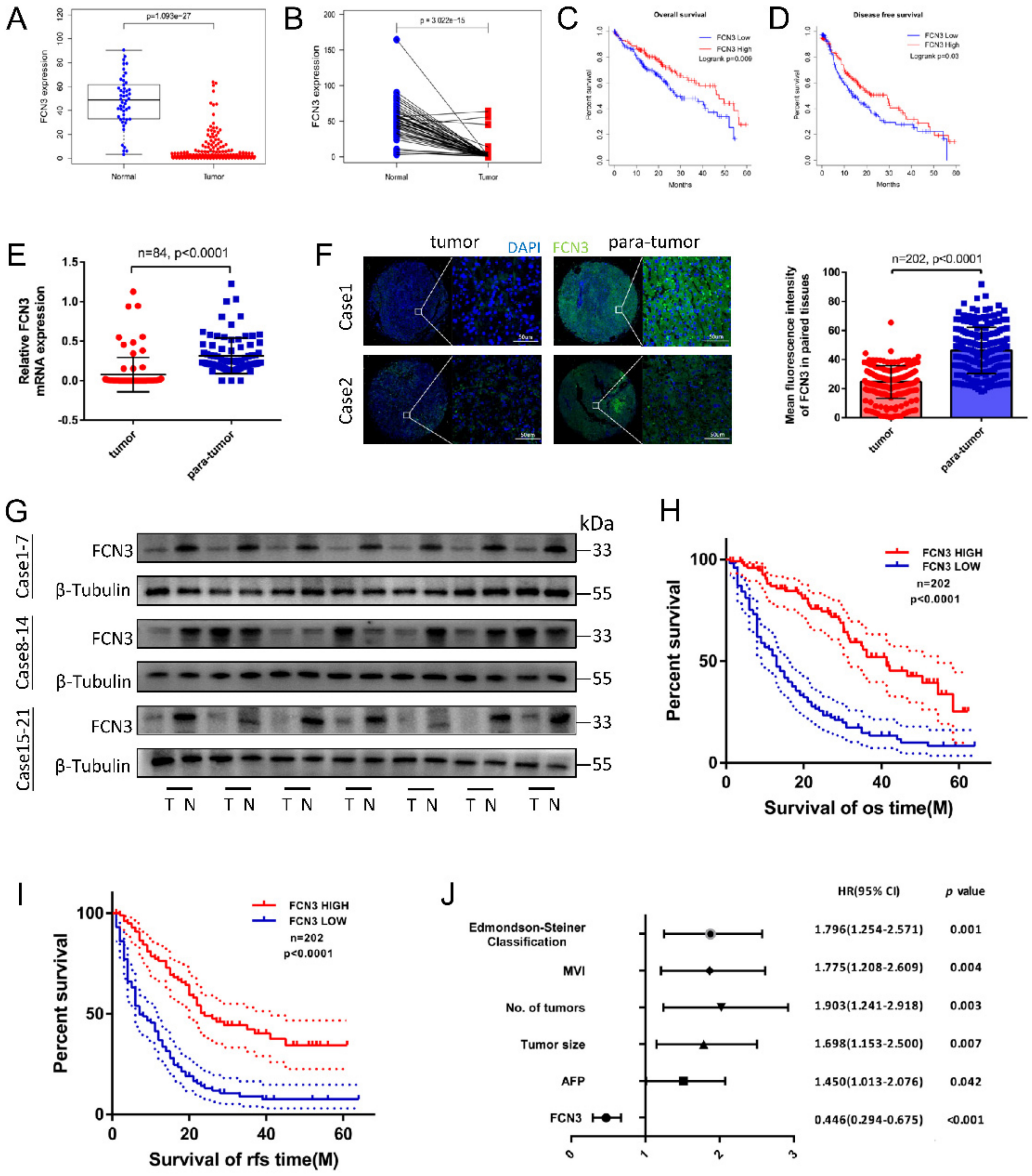
** Low expression of FCN3 correlated with poor prognosis. (A)** FCN3 expression in 50 normal samples and 374 tumor samples from TCGA.** (B)** FCN3 expression in 50 pairs of HCC samples from TCGA. **(C, D)** Kaplan-Meier survival curves of OS and DFS of 362 patients from TCGA. **(E)** FCN3 mRNA expression was downregulated in tumor tissues compared to 84 paired para-tumor tissues by qRT-PCR. **(F)** Representative images of IF and the mean fluorescence intensity of FCN3 in 202 paired tumor and para-tumor tissues. **(G)** FCN3 protein expression was showed in 24 paired tumor and para-tumor tissues by Western blot. (“T” for tumor, “N” for non-tumor). **(H, I)** Kaplan-Meier survival curves of OS and RFS time for 202 HCC patients. **(J)** Forest plot of the multivariate Cox regression analysis.

To investigate the pathogenic and clinical significance of FCN3 in HCC, we first analyzed the expression pattern of FCN3 using The Cancer Genome Atlas (TCGA) database. The results showed that the expression level of FCN3 was downregulated in various cancers (Supplementary file 2: Fig. S1A). In liver hepatocellular carcinoma (LIHC) cohort, the expression of FCN3 was significantly downregulated in HCC tissues compared to unpaired or paired liver tissues (Fig. 1A, 1B). Moreover, patients with low expression of FCN3 exhibited shorter overall survival (OS) and disease-free survival (DFS) (Fig. 1C, 1D). The expression of FCN3 was verified in multiple cohorts from the Oncomine database and identical results were obtained (Supplementary file 2: Fig. S1B-E).

We further examined the transcriptional level of FCN3 in paired tumor vs non-tumor samples by qRT-PCR. The results showed that the mRNA expression of FCN3 in HCC tissues was significantly decreased compared with the adjacent normal liver tissues (Fig. 1E). The protein expression levels of FCN3 were consistent with the mRNA levels, as evidenced by the immunofluorescence and Western blot (Fig. 1F, 1G). In addition, Kaplan-Meier survival analysis revealed that low expression of FCN3 was significantly associated with poor prognosis. Patients with low FCN3 expression level had a significantly worse OS and recurrence-free survival (RFS) than those who with high FCN3 expression (Fig. 1H, 1I). Clinical data analysis indicated that low expression of FCN3 was significantly correlated with AFP, tumor size, number of tumors, microvascular invasion and Edmondson-Steiner classification in 202 patients of HCC (Table 1). To further verify the robustness value of FCN3 expression, multivariate analysis was performed to determine risk assessment related to OS. The results confirmed FCN3 as an independent unfavorable prognostic factor of HCC (Fig. 1J and Supplementary file 1: Table S1).

### 3.2. FCN3 overexpression promoted apoptosis and suppressed proliferation in hepatoma cells

**Figure 2:**
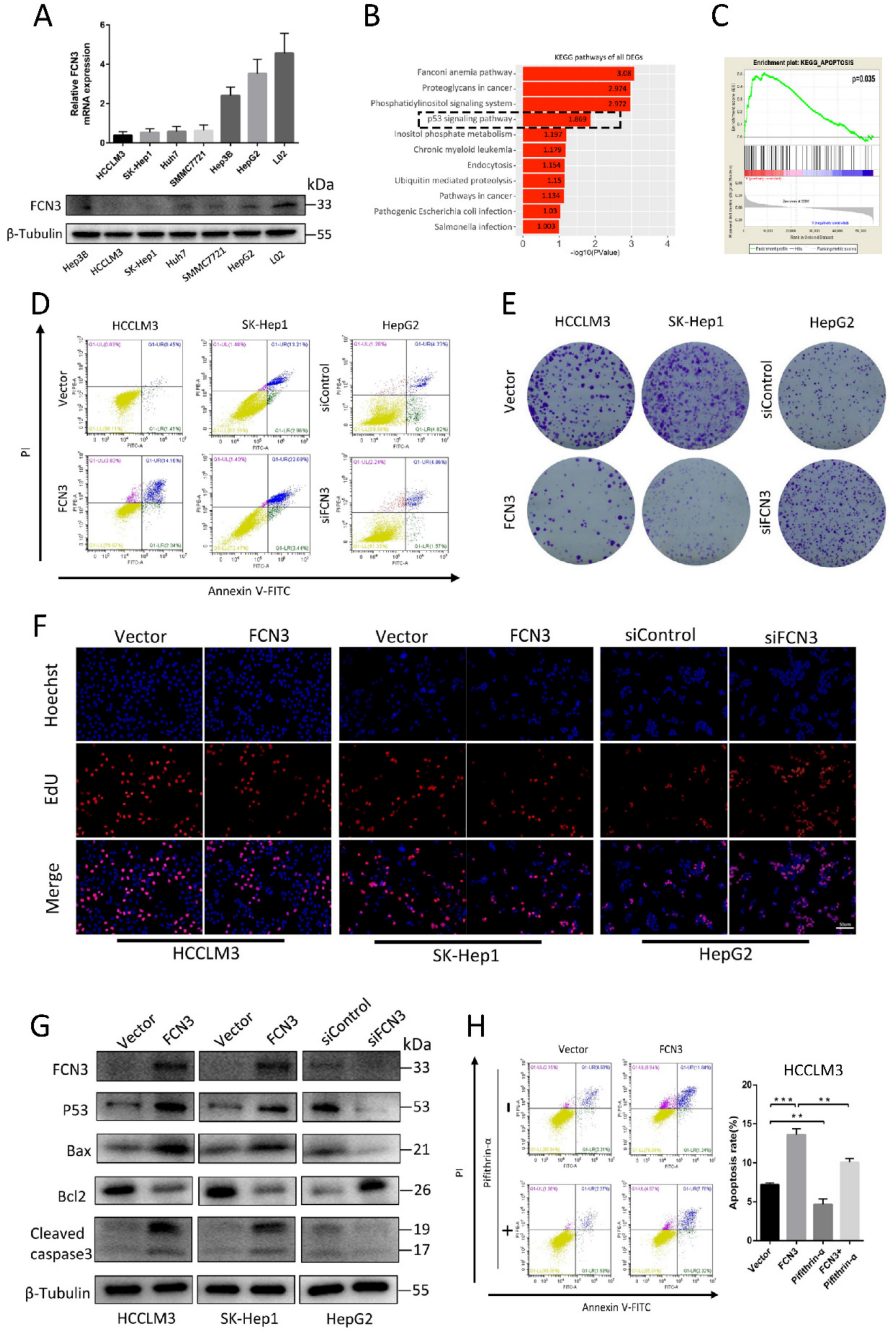
** Overexpression of FCN3 induced apoptosis and inhibited cell proliferation. (A)** FCN3 mRNA and protein level in HCC cells and normal liver cell L02. **(B)** KEGG pathway analysis of all DEGs in cell transcriptome sequencing and the p53 signaling pathway shown by the black dotted frame. **(C)** Single-gene GSEA analysis of FCN3 with the TCGA data.** (D)** FCN3 overexpression in HCCLM3 and SK-Hep1 increased apoptosis whereas FCN3 knockdown decreased apoptosis of HepG2 cells. **(E)** FCN3 overexpression inhibited cell proliferation in HCCLM3 and SK-Hep1, silenced FCN3 promoted cell proliferation in HepG2. **(F)** Representative images of EdU positive cells from an EdU incorporation assay. **(G)** Western blot analysis showing expression of FCN3, p53, Bax, Bcl2, and Cleaved caspase3 in HCCLM3 and SK-Hep1 transfected with FCN3 and HepG2 transfected with siFCN3. **(H)** Apoptosis rate of HCCLM3 cells overexpression FCN3 and treated with Pifithrin-α (p53 inhibitor, 30uM, 24h) as determined by flow cytometry. Results are expressed as the mean±SD of three independent experiments. **P<0.01, ***P<0.001.

To determine the role of FCN3 in HCC, we detected the expression levels of FCN3 in various HCC cell lines and normal liver cell line L02. Results of qRT-PCR and Western blot suggested the expression level of FCN3 in HCC cell lines was lower in both mRNA and protein levels compared with L02. (Fig. 2A). FCN3 was overexpressed in HCCLM3 and SK-Hep1 cells (Supplementary file 2: Fig. S2A, S2C), and knocked down in HepG2 cells by transfecting plasmids and siRNAs separately (Supplementary file 2: Fig. S2B, S2C). We performed cell transcriptome sequencing after FCN3 and vector transfection in HCCLM3 cells (Supplementary file 3: TXT 1). We defined the genes with fold change ≥2 or ≤0.5, *P* < 0.05 (log2(fold change) ≥1 or ≤-1, -log10(*P*) > 1.3) in the transcriptome sequencing results as differentially expressed genes (DEGs). KEGG enrichment analysis of DEGs in cell transcriptome sequencing showed that FCN3 upregulation was associated with the p53 signaling pathway (Fig. 2B), which was widely reported to be involved in cell proliferation, apoptosis (20-22). Meanwhile, Gene set enrichment analysis (GSEA) was performed with TCGA data and the results indicated that the high expression of FCN3 was closely related to apoptosis (Fig. 2C). The biological function of FCN3 in HCC was next investigated by flow cytometry, colony formation assay and EdU assay. Flow cytometry results showed that apoptotic cells increased significantly when FCN3 was overexpressed, while decreased when FCN3 was knocked down (Fig. 2D, Supplementary file 2: Fig. S2D, S2E). Results of colony formation assay showed that FCN3 overexpression inhibited cell proliferation while FCN3 downregulation promoted cell proliferation (Fig. 2E, Supplementary file 2: Fig. S2F, S2G). The same trend was confirmed in the EdU assay (Fig. 2F, Supplementary file 2: Fig. S2H, S2I, S2J). Meanwhile, Western blot assay evidenced FCN3 was closely related to p53-mediated apoptosis. Overexpression of FCN3 in HCCLM3 and SK-Hep1 increased the protein expression of p53, Bax, Cleaved caspase3 and reduced protein expression of Bcl2. Knockdown of FCN3 in HepG2 got the opposite results (Fig. 2G). To further verify whether FCN3 overexpression promoted p53-mediated apoptosis, we detected the apoptosis rate of HCCLM3 cells overexpressed FCN3 and treated with Pifithrin-α (p53 inhibitor). As shown in Figure 2H, Pifithrin-α attenuated FCN3-induced apoptosis in HCCLM3 cells. These results indicate that FCN3-induced apoptosis is associated with p53.

### 3.3. The interaction of FCN3 with SBDS reduced the nuclear import of EIF6 and induced ribosome stress

**Figure 3:**
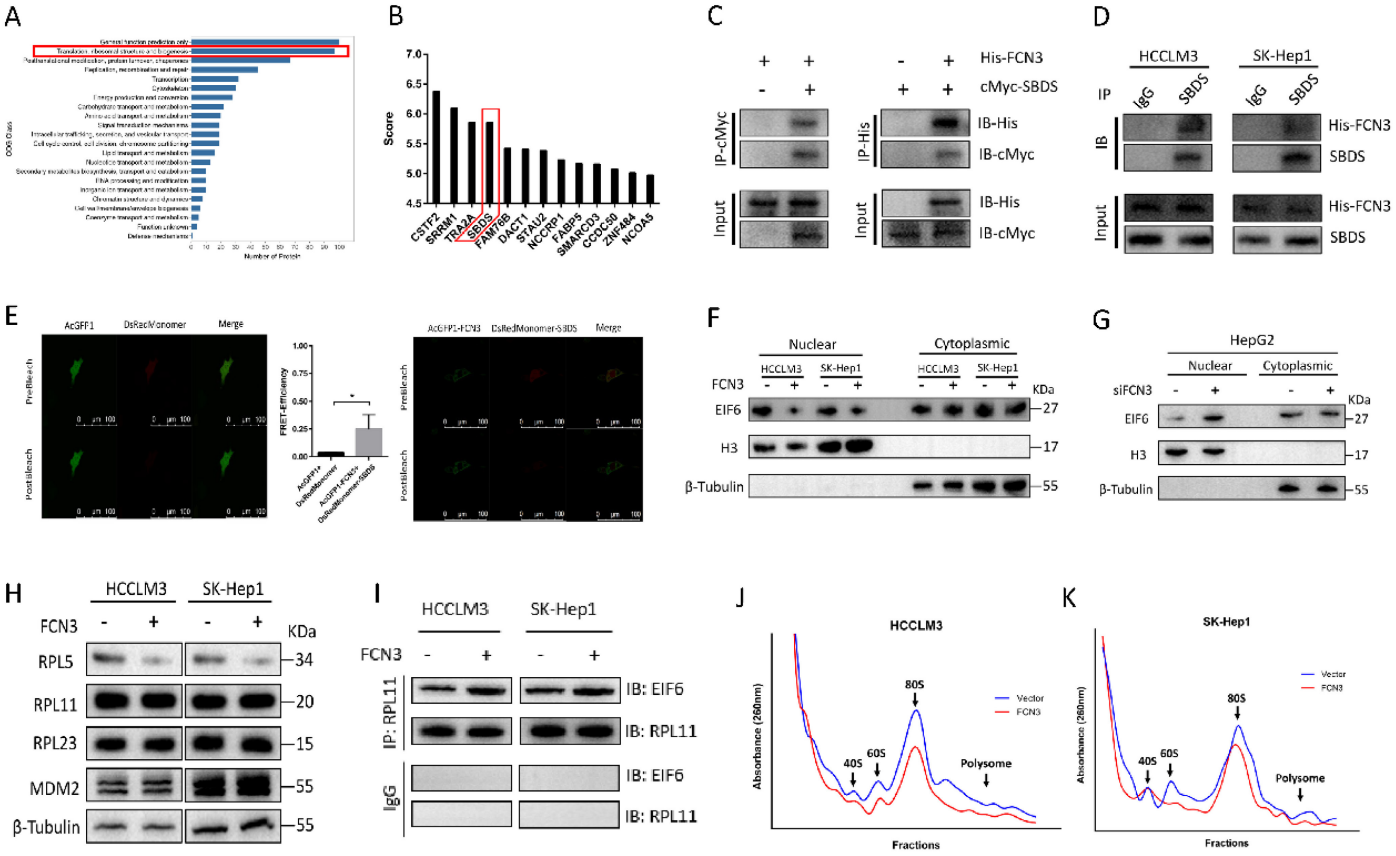
** FCN3 interacted with SBDS and regulated the nuclear import of EIF6 and ribosome biogenesis. (A)** COG class annotation results of all identified proteins by Co-IP MS assay. **(B)** FCN3-binding proteins in the Co-IP MS results and top 13 score of proteins were shown. **(C)** Exogenous Co-IP assay in HEK-293T cells co-transfected with His-FCN3 and cMyc-SBDS. **(D)** Endogenous Co-IP assay in HCCLM3 and SK-Hep1 cells transfected with His-FCN3. **(E)** FRET assay in living HCCLM3 cells (FRET-Efficiency= (Donor_post_-Donor_pre_)/ Donor_post_). **(F)** Western blot of EIF6 nucleocytoplasmic separation in HCCLM3 and SK-Hep1 overexpressing FCN3. **(G)** Western blot of EIF6 nucleocytoplasmic separation in HepG2 cells downregulated FCN3. **(H)** Western blot of RPL5, RPL11, RPL23 and MDM2 in HCCLM3 and SK-Hep1 cells transfected FCN3. **(I)** Co-IP assay with RPL11 antibody in HCCLM3 and SK-Hep1 cells overexpressed FCN3. **(J, K)** Ribosome profiles at 260nm absorbance in HCCLM3 and SK-Hep1 cells overexpressing FCN3. The location of 40S, 60S and 80S were indicated by arrows. *P<0.05.

To gain mechanistic insight into FCN3 function in HCC, Co-IP MS was utilized to analyze proteins which may interact with FCN3. HCCLM3 cells transfected with His-FCN3 were subjected to Co-IP assay with His-tag antibody, and protein samples eluted from the magnetic beads were used for MS identification. Clusters of Orthologous Groups (COG) functional annotation of all identified proteins showed a large number of proteins were involving in ribosome biogenesis (Fig. 3A). Top20 KEGG pathway annotation also revealed that 54 proteins in the Co-IP MS results were associated with cell ribosome related processes (Supplementary file 2: Fig. S3A). Therefore, we examined the proteins in the Co-IP MS results, among which the ribosome maturation factor SBDS attracted our attention (Fig. 3B). To verify the interaction between FCN3 and SBDS, His-FCN3 and cMyc-SBDS were transfected in HEK-293T cells with His-tag antibody and Myc-tag antibody for exogenous Co-IP assay (Fig. 3C). Moreover, endogenous Co-IP assay with IgG and SBDS antibody in HCCLM3 and SK-Hep1 cells overexpressing His-FCN3 showed His-FCN3 were present in SBDS immune complex, but not in IgG immune complex (Fig. 3D). Then, we confirmed the binding of FCN3 and SBDS in living cells by fluorescence resonance energy transfer (FRET) experiment by co-transfection of AcGFP1-FCN3 and DsRedMonomer-SBDS in HCCLM3 cells (Fig. 3E).

According to previous study, SBDS triggered the release of EIF6 from the 60S ribosome subunit in the cytoplasm, enhanced its nuclear translocation, thereby participated in the biogenesis and maturation of ribosomes 23. Impaired ribosome biogenesis induces ribosome stress and inhibits ubiquitination degradation of p53, resulting in accumulation of p53 in cells and cell apoptosis 24. Thus, we hypothesized that FCN3 exerted its function by regulating the above process. Protein extracted from the nucleus and cytoplasm fractions and subjected to immunoblotting, we found that EIF6 in the nucleus was significantly reduced after overexpression of FCN3 (Fig. 3F). Cell immunofluorescence (IF) also suggested the same conclusion (Supplementary file 2: Fig. S3D). In contrast, EIF6 in the nucleus was increased when FCN3 was knocked down (Fig. 3G). However, the mRNA level and total protein level of EIF6 in the cells showed no obvious change when upregulating FCN3 (Supplementary file 2: Fig. S3C). Furthermore, we detected the protein levels of MDM2 and ribosomal proteins, including RPL5, RPL11 and RPL23. Western blot results showed that RPL5 protein was decreased with overexpression of FCN3, however, the expression of RPL11, RPL23 and MDM2 was not significantly (Fig. 3H). Subsequently, ribosomes isolated by sucrose gradient were employed for Co-IP assay. Co-IP assay with RPL11 antibody was used to analyze the changes of EIF6 protein on the ribosome, and the results indicated a significant increase of EIF6 on the ribosome when FCN3 was overexpressed (Fig. 3I). These results also further demonstrated that FCN3 affected the nuclear import of EIF6 by interacting with SBDS, instead of regulating the total EIF6 levels. To explore whether the interaction of FCN3 and SBDS affects the ribosome biogenesis, HCCLM3 and SK-Hep1 cells were firstly transfected with vector or FCN3, and cell lysates were subjected to sucrose density gradient centrifugation. The ribosome profiles confirmed a significant decrease in 60S subunit, compared to 40S, and a concomitant reduction in 80S in cells overexpressing FCN3 (Fig. 3J, 3K).

### 3.4. YBX1 participates in FCN3-induced SBDS downregulation

**Figure 4:**
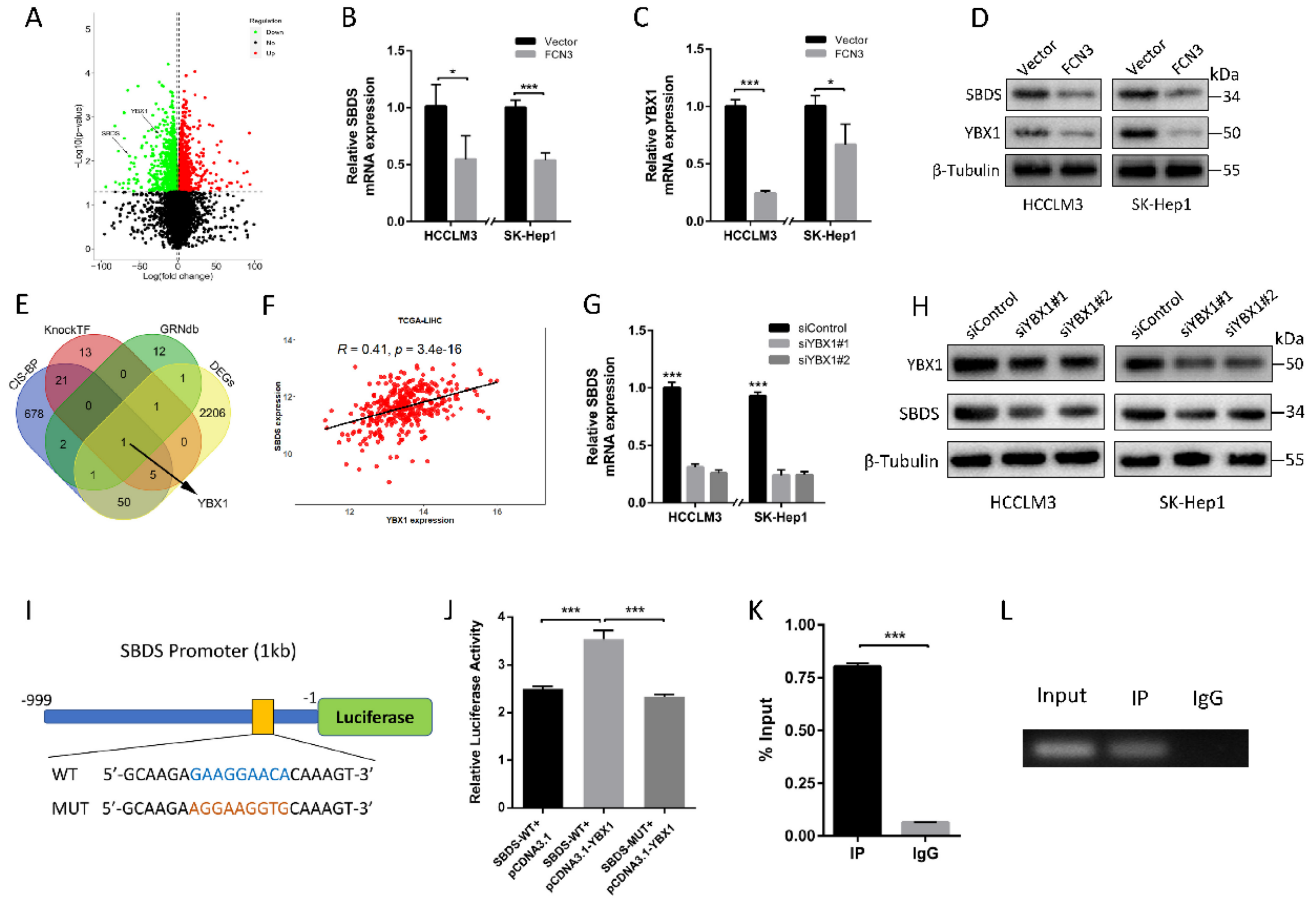
** YBX1 contributed to the regulation of FCN3 on SBDS. (A)** Volcano map of DEGs in transcriptome sequencing. SBDS and YBX1 were marked by two black arrows respectively. **(B)** Relative SBDS mRNA expression in cells overexpressing FCN3 as determined by qRT-PCR. **(C)** Relative YBX1 mRNA expression in cells overexpressing FCN3 as determined by qRT-PCR. **(D)** Western blot of SBDS and YBX1 after FCN3 overexpression. **(E)** Venn plots of CIS-BP、KnockTF、GRNdb database and DEGs. **(F)** Correlation analysis of SBDS and YBX1 in TCGA-LIHC dataset. **(G)** Relative SBDS mRNA expression in YBX1 knockdown cells. **(H)** SBDS protein expression in YBX1 knockdown cells. **(I)** Schematic diagram of YBX1 binding site on the WT and MUT SBDS promoter. **(J)** Relative luciferase activity of SBDS WT and MUT promoter in HEK-293T cells. **(K)** ChIP-PCR assay was performed with PCR primers of SBDS promoter (%Input = 2% * 2^(CT^Input^ - CT^sample^). **(L)** Agarose gel electrophoresis of the ChIP-PCR products. Each histogram is the mean±SD of three independent experiments. *P<0.05, ***P<0.001.

It is interesting to note that FCN3 downregulated the expression of SBDS based on our transcriptome sequencing results (Fig. 4A). In further analyses, we preformed qRT-PCR and Western blot to verify the effect of FCN3 on mRNA and protein levels of SBDS (Fig. 4B, 4D). To investigate the mechanism of FCN3 on SBDS expression occurred at the transcriptional level, potential transcription factors (TFs) involved were screened. Intersection of predicted TFs from CIS-BP, KnockTF, GRNdb database and DEGs was taken. YBX1, a TF can significantly affect tumor development, invasion, metastasis, and other malignant biological behaviors 25, 26, aroused our interest (Fig. 4E). Interestingly, YBX1 was also confirmed regulating by FCN3 (Fig. 4C, 4D). Bioinformatics analysis of TCGA-LIHC showed a positive correlation between SBDS and YBX1 expression (Fig. 4F), this relationship suggests that there may be a regulatory relationship between the two. In addition, the results of qRT-PCR and Western blot after YBX1 knockdown indicated that YBX1 was positively regulated SBDS (Fig. 4G, 4H). Luciferase reporter vectors containing SBDS WT or MUT promoter were constructed for further verification (Fig. 4I). As a result, overexpression YBX1 significantly increased the luciferase activity of the WT vector compared with the MUT (Fig. 4J). Importantly, ChIP-PCR confirmed the binding of YBX1 to the SBDS promoter (Fig. 4K). Agarose gel electrophoresis was performed to analyze the products of ChIP-PCR, and IP group revealed strong enrichment relative to IgG group (Fig. 4L).

### 3.5. YBX1 diminished the tumor suppressive effect induced by FCN3

**Figure 5:**
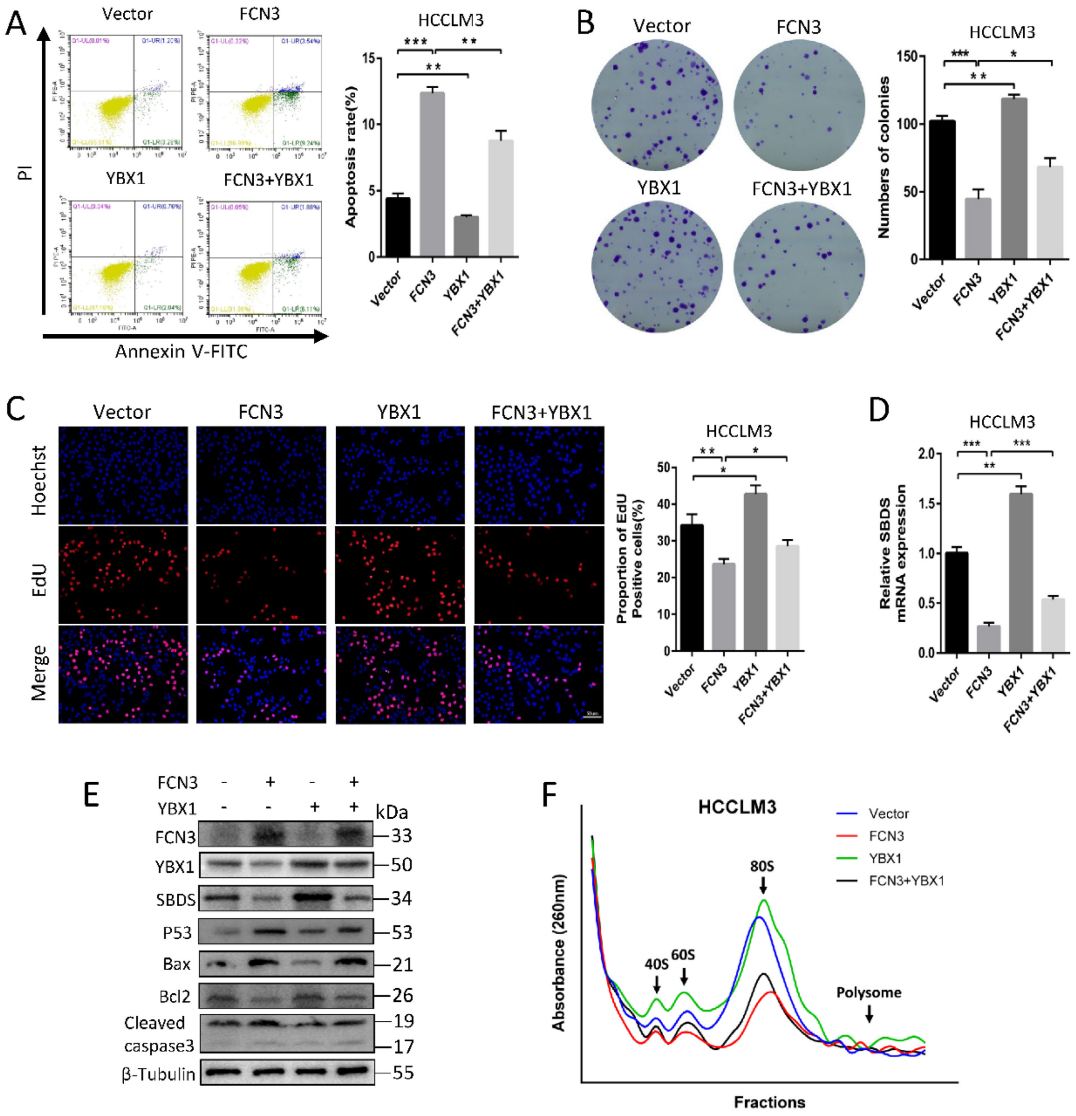
** YBX1 rescued the FCN3-induced tumor inhibition. (A)** Apoptosis rate of HCCLM3 cells transfected FCN3 and YBX1 by flow cytometry. **(B)** Colony formation assay of HCCLM3 cells was performed to examine the effect on cell proliferation. **(C)** EdU incorporation assay of HCCLM3 cells transfected FCN3 and YBX1. **(D)** Relative SBDS mRNA expression in HCCLM3 cells co-transfected with FCN3 and YBX1. **(E)** Effect of FCN3 and YBX1 overexpression on SBDS and p53-mediated proteins levels. **(F)** Ribosome profiles at 260nm absorbance in HCCLM3 cells overexpressing FCN3 and YBX1. The location of 40S, 60S and 80S were indicated by arrows. Histograms represent the mean±SD of three independent experiments. *P<0.05, **P<0.01, ***P<0.001.

Based on the above regulation of FCN3 on YBX1 and SBDS, the effect of YBX1 on FCN3-induced functional consequences was investigated. Flow cytometry results showed that FCN3 and YBX1 overexpressing reduced cell apoptosis rate in cells compared with cells overexpressing FCN3 only (Fig. 5A). Meanwhile, co-transfection of FCN3 and YBX1 increased the numbers of colonies compared with the FCN3 group (Fig. 5B). EdU results also revealed that simultaneous overexpression of YBX1 inhibited the effect of FCN3 upregulation on cell proliferation (Fig. 5C). In addition, flow cytometry results of Nutlin-3-treated HCCLM3 cells showed that YBX1 inhibited apoptosis and YBX1 overexpression reduced Nutlin-3-induced apoptosis (Supplementary file 2: Fig. S4A). Furthermore, we demonstrated YBX1 impaired FCN3-induced downregulation of SBDS on mRNA and protein levels. (Fig. 5D, 5E). Moreover, analysis of proteins associated with apoptosis, such as p53, Bax, Bcl2, Cleaved caspase3, revealed that YBX1 diminished the tumor suppressor function of FCN3 (Fig. 5E). The ribosome profiles showed that YBX1 overexpression significantly enhanced the ribosome biogenesis, and YBX1 alleviated FCN3-induced ribosome stress (Fig. 5F).

### 3.6. FCN3 inhibited tumor growth and induced cell apoptosis *in vivo*

**Figure 6:**
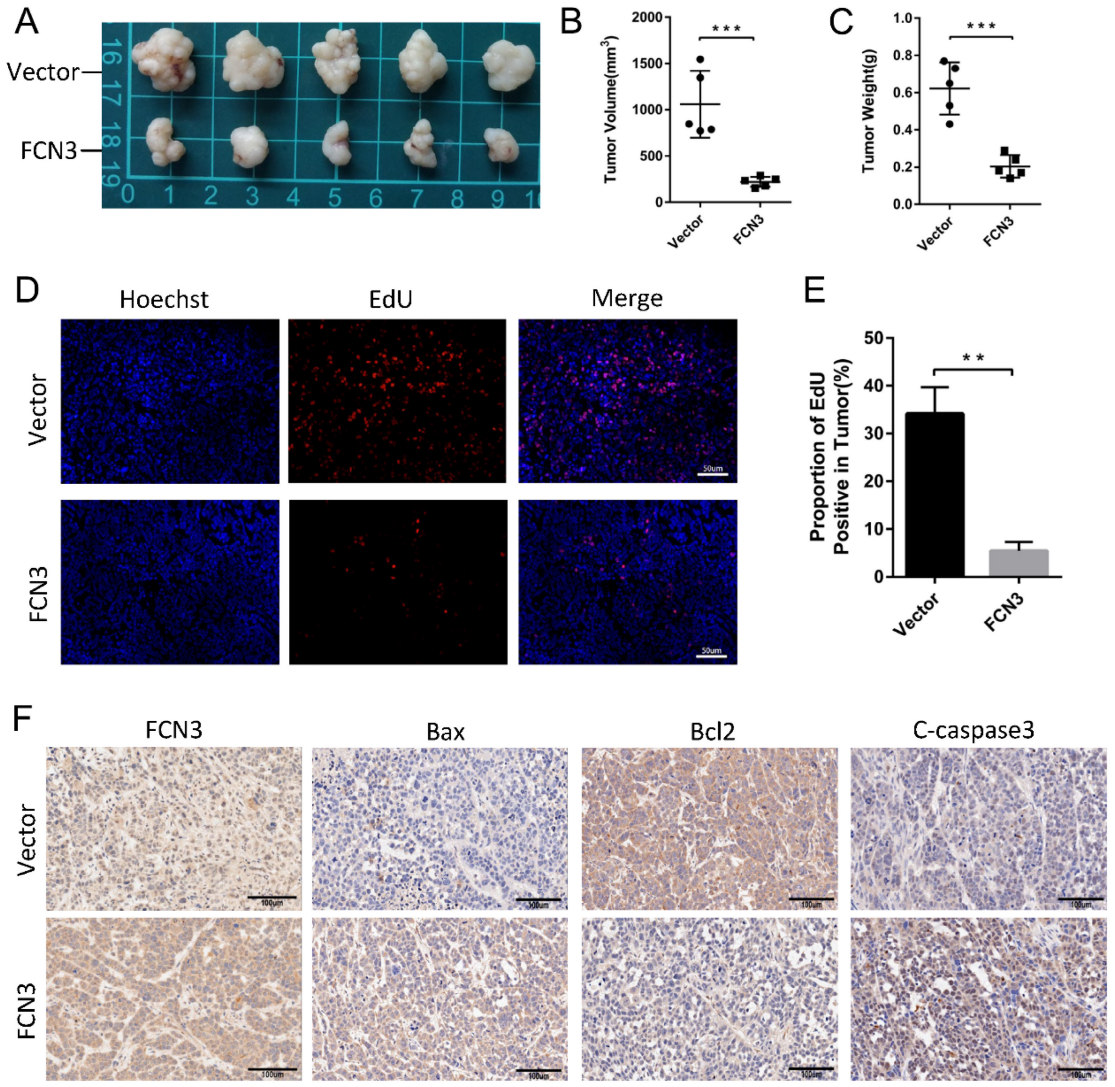
** The influence of FCN3 on HCC *in vivo*. (A)** The subcutaneous xenograft models were established in nude mice. **(B, C)** The volume and weight of subcutaneous xenograft tumors between the control group and the FCN3 group. **(D, E)** EdU incorporation assay of tumor tissue sections. **(F)** IHC of Bax, Bcl2 and cleaved caspase-3 expression in FCN3 and control group. The histograms indicate mean±SD from three independent experiments. **P<0.01, ***P<0.001.

The subcutaneous xenograft model was constructed by subcutaneous injection of HCCLM3 cells. We discovered that FCN3 overexpression significantly reduced the volume of subcutaneous xenograft tumors and restrained tumor growth (Fig. 6A, 6B, 6C). The proportion of EdU positive cells in tumor tissues was confirmed, and a significant decrease in the FCN3 group was observed compared to the control group (Fig. 6D, 6E). Immunohistochemistry (IHC) showed a decreased expression of Bcl2, and increased expression of Bax and Cleaved caspase-3 in tumors from FCN3 group compared with those from the control group, which indicated FCN3 enhanced activation of apoptosis pathway *in vivo* (Fig. 6F).

### 3.7. Negative feedback regulation of p53 on YBX1/SBDS in the downstream of FCN3

**Figure 7:**
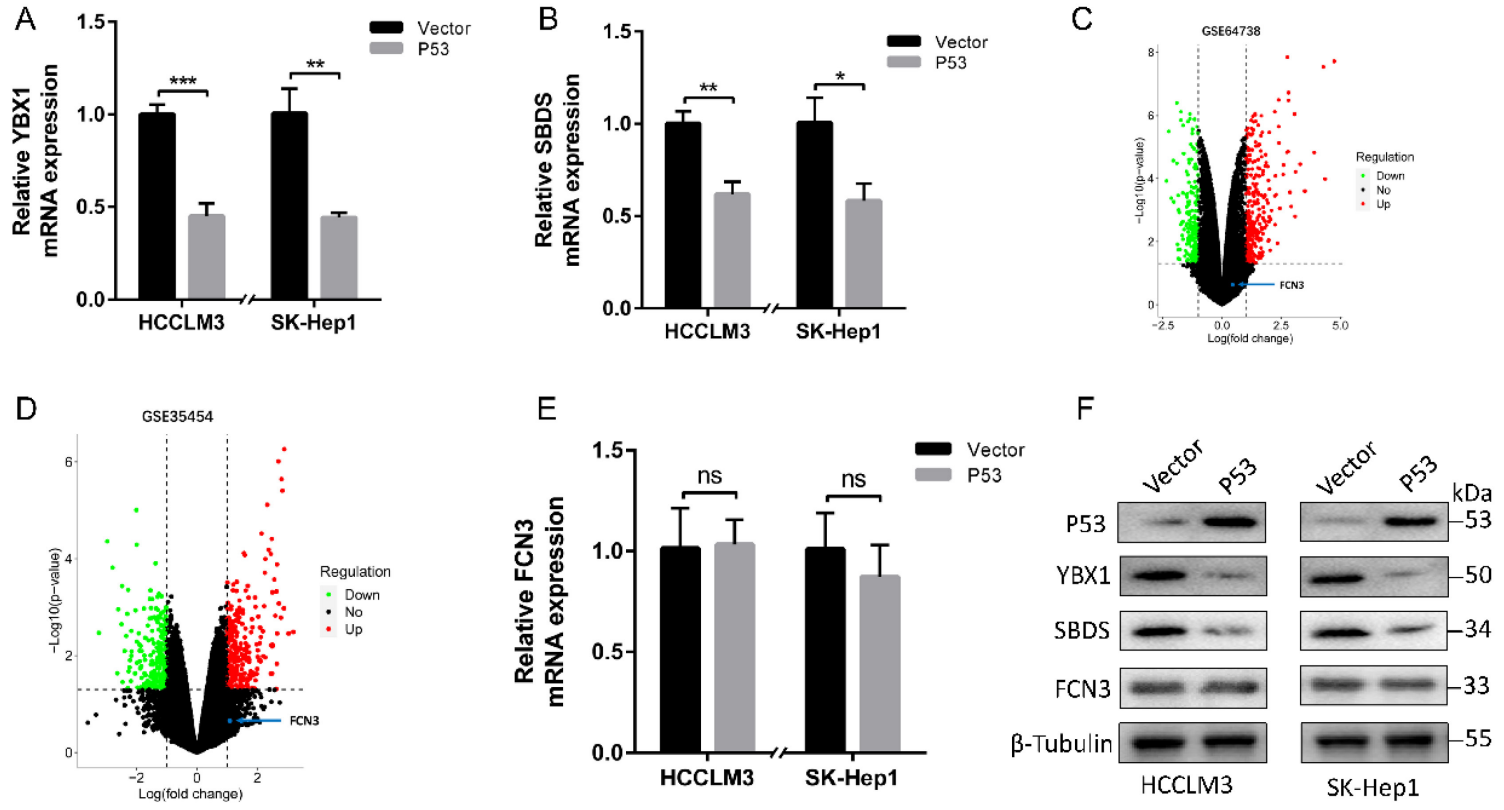
** Negative feedback regulation of p53 on YBX1/SBDS. (A, B)** Relative YBX1 and SBDS mRNA expression in HCCLM3 and SK-Hep1 cells when WT p53 increased. **(C)** The volcano maps of differential expressed genes in GSE64738 (Pleural mesothelioma cells with p53 overexpression). **(D)** The volcano maps of differential expressed genes in GSE35454 (Human glioblastoma cells with p53-specific siRNA). The position of FCN3 was shown by blue point and arrow. **(E)** Relative FCN3 mRNA expression in HCCLM3 and SK-Hep1 cells overexpressing WT p53. **(F)** YBX1, SBDS and FCN3 protein levels when WT p53 was overexpressed in HCCLM3 and SK-Hep1 cells. The results represent the mean±SD of three independent experiments. *P<0.05, **P<0.01, ***P<0.001.

To generalize the preceding findings, we conclude that FCN3 regulates the expression of SBDS via YBX1 and interacted with SBDS to induce ribosome stress, thus eventually lead to the increase and accumulation of p53 in hepatoma cells. As a tumor suppressor and transcription factor, p53 plays an important role in many biological processes like apoptosis, cell cycle and DNA repair 27. Next, we investigated the associations among FCN3, YBX1, SBDS and p53 through literatures review. At least two different mechanisms for the regulation of YBX1 by p53 were reported 28. Consistent with previous literatures, the expression of YBX1 and SBDS was downregulated following overexpression of wild type (WT) p53 in HCCLM3 and SK-Hep1 cells (Fig. 7A, 7B, 7F). The volcano maps of GSE64738 and GSE35454 revealed that there was no significant difference in expression of FCN3 following either p53 overexpression or knockdown (Fig. 7C, 7D). In addition, the mRNA and protein level of FCN3 did not vary significantly when WT p53 was overexpressed in HCCLM3 and SK-Hep1 cells (Fig. 7E, 7F). Therefore, a negative feedback regulation of p53 on YBX1/SBDS were conducted.

## 4. Discussion

FCN3 protein is a member of the human ficolin family which consist of an N-terminal domain, a collagen-like domain and a fibrinogen-like domain 29. It is secreted into the bile duct and blood circulation once produced by hepatocytes and bile duct epithelial cells in liver 30. Previous studies have demonstrated that FCN3 is a potential serum biomarker in HCC patients, especially among patients infected with HCV 13. However, the influence of FCN3 on the overall prognosis and cancer biology of HCC are not well characterized. Here, we found that FCN3 expression was significantly downregulated in hepatoma cells and HCC tissues on both mRNA and protein level and that FCN3 function as a tumor suppressor. OS and RFS analysis revealed that low expression of FCN3 was significantly associated with poor prognosis. Furthermore, clinical data analysis confirmed that FCN3 was an independent unfavorable prognostic factor of HCC. Functionally, FCN3 promoted apoptosis and suppressed proliferation *in vitro* and *in vivo*. Mechanistically, FCN3 interacted with SBDS and regulated the nuclear import of EIF6, resulted in ribosomal stress. In addition, YBX1 involved in the transcription and translation level regulation of FCN3 to SBDS. Besides, a negative feedback loop in the downstream of FCN3 involving p53, YBX1 and SBDS was identified.

SBDS protein is required for the assembly of mature ribosomes and ribosome biogenesis. It facilitates the release of EIF6, a key protein in preventing the association of the 40S and 60S ribosomal subunits, from 60S ribosomal subunits. SBDS enhances the nuclear translocation of EIF6 in cryo-EM study 31. In this study, we found that FCN3 interacts with SBDS involving in ribosome stress and p53 activation. We investigated the effect of FCN3 on the regulation of ribosomal stress. The interaction of FCN3 and SBDS reduced the nuclear import of EIF6 and induced ribosome stress. Proteins nucleocytoplasmic separation experiment and IF showed that EIF6 in the nucleus was significantly reduced when FCN3 overexpressed, but no evident changes were observed in EIF6 expression at transcriptional and translational levels. Ribosome profiling also supported the deficiency of ribosome biogenesis. Here, we for the first time reported FCN3 participated in the SBDS/EIF6 associated ribosome stress.

Remarkably, overexpressed FCN3 performed significant downregulation of SBDS in our transcriptome sequencing results. YBX1 was downregulated by FCN3 and was considered a positive transcription factor of SBDS through bioinformatics analysis. High YBX1 expression contributes to malignant progression, and closely associates with sorafenib resistance in HCC 32-34. Here we for the first time reported YBX1 involving in the development of HCC through a role of broker between FCN3 and SBDS. However, the regulation mechanism of FCN3 on YBX1 is still unclear and needs further study.

**Figure 8:**
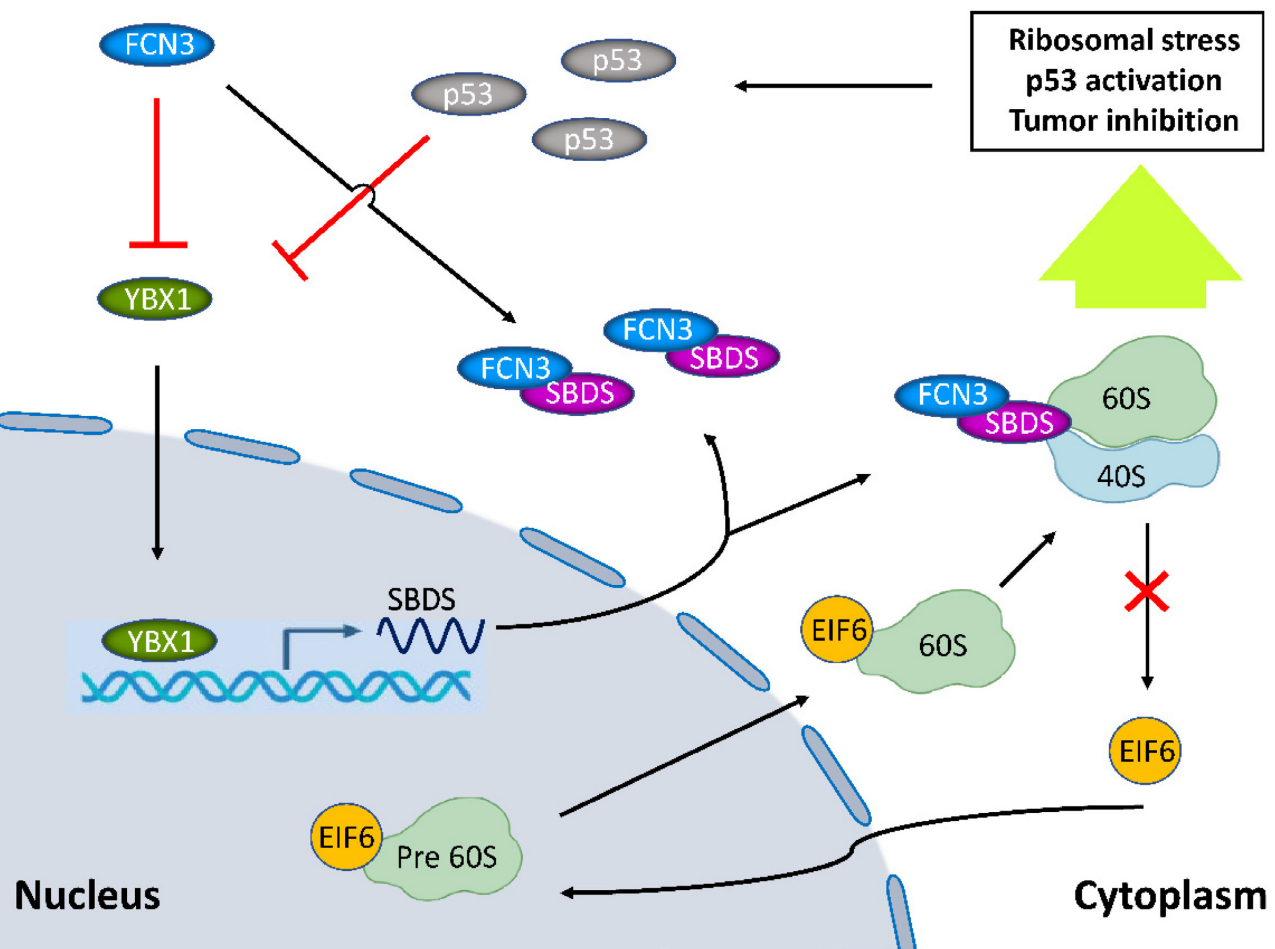
** Model diagram showing the inhibitory effect of FCN3 on tumor progression in HCC cells.** FCN3 suppressed the expression level of SBDS by downregulating YBX1. The interaction between FCN3 and SBDS reduced the nuclear import of EIF6, induced ribosomal stress and activation of the p53 pathway. A negative feedback regulation of p53 on YBX1/SBDS in the downstream of FCN3.

Transcriptome sequencing and the GSEA results showed that FCN3 was significantly associated with p53 signaling pathway and apoptosis. The p53 signaling pathway is generally activated by diverse stress signals, including DNA damage 17, hypoxia 35 and Nitric oxide 36 as well as oncogenes activation 37-40. Zambetti Noemi A. *et al*. 41 reported SBDS deficiency activated the p53 tumor suppressor pathway and induced apoptosis in late-stage myeloid cells. In our study, we identified FCN3 regulating p53 pathway through a SBDS-dependent manner. Meanwhile, the accumulation and activation of p53 was considered a response to ribosomal stress, because a number of ribosome proteins entered the nucleoplasm to interact with MDM2, resulting in p53-mediated apoptosis in this stress 42, 43. Moreover, it has been reported that YBX1 is a downstream gene of p53 which can depress YBX1 by regulating mTOR or miR34a 28. Our data also confirmed p53-mediated downregulation of YBX1 leaded to a subsequent decrease of SBDS, and here we reported a negative feedback regulation of p53 on YBX1/SBDS in the downstream of FCN3 (Fig. 8).

In summary, this study clarified the biological function of FCN3 in hepatoma cells. Besides, this is the first to demonstrate that FCN3 leads to the dissonance in SBDS expression and function. These findings would extend and deepen our understanding of the role of FCN3 and SBDS in HCC, and it is suggested the regulation of the FCN3/YBX1/SBDS axis may be a novel strategy for the treatment of HCC.

## Supplementary Material

Supplementary figures and tables.

## Figures and Tables

**Table 1:** Association of FCN3 expression with clinicopathological characteristics of HCC patients

Characteristics	FCN3 expression		Number of patients	*p* value
	High (n=101)	Low (n=101)		
**Age(y)**				0.596
≤55	64	71	135	
>55	37	30	67	
**Gender**				0.221
Male	88	92	180	
Female	13	9	22	
**HBV DNA load (IU/ml)**				0.163
≤10^4^	75	57	132	
>10^4^	26	44	70	
**AFP (ng/ml)**				0.004*
≤400	64	44	108	
>400	37	57	94	
**ALB (g/l)**				0.163
≤40	40	49	89	
>40	61	52	113	
**Liver cirrhosis**				0.285
Yes	61	53	114	
No	40	48	88	
**Tumor size (cm)**				<0.001*
≤5	59	27	86	
>5	42	74	116	
**No. of tumors**				<0.001*
Solitary	88	73	161	
Multiple	13	28	41	
**MVI**				<0.001*
Presence	21	37	58	
Absence	80	64	144	
**Edmondson-Steiner classification**				0.005*
I-II	46	47	93	
III-IV	55	54	109	

* Significant results (*P* < 0.05) are given in bold. Abbreviation: HBV, hepatitis B virus; MVI, microvascular invasion.

## Abbreviations

HCC: Hepatocellular carcinoma
FCN3: Ficolin-3
SBDS: Ribosome Maturation Factor
EIF6: Eukaryotic Translation Initiation Factor 6
YBX1: Y-Box Binding Protein 1
TCGA: The Cancer Genome Atlas
GSEA: gene set enrichment analysis
KEGG: Kyoto Encyclopedia of Genes and Genomes
OS: overall survival
DFS: disease-free survival
RFS: recurrence-free survival
AFP: Alpha Fetoprotein
EdU: 5-Ethynyl-2′-deoxyuridine
DEGs: differentially expressed genes
COG: Clusters of Orthologous Groups
FRET: Fluorescence resonance energy transfer
IHC: Immunohistochemistry
IF: Immunofluorescence
ChIP: Chromatin immunoprecipitation
