# Supplementary material for: FCN3 inhibits the progression of hepatocellular carcinoma by suppressing SBDS-mediated blockade of the p53 pathway
Source: Int J Biol Sci. 2023 Jan 1;19(2):362–76. doi: 10.7150/ijbs.69784 (PMC9830510; doi:10.7150/ijbs.69784)
Supplement: Supplementary file 1 — Supplementary figures and tables. [file ijbsv19p0362s1.pdf]

Table S1: Univariate and multivariate analysis with a Cox proportional hazard regression model for overall survival.

variable	Univariate Analysis			Multivariate Analysis		
	**HR**	**95% CI**	***p* value**	**HR**	**95% CI**	***p* value**
Age	1.103	0.768-1.585	0.596	-	-	-
Gender	0.680	0.366-1.262	0.221	-	-	-
HBV DNA load	1.289	0.902-1.842	0.163	-	-	-
ALB	0.780	0.550-1.106	0.163	-	-	-
Liver cirrhosis	0.827	0.583-1.172	0.285	-	-	-
Grade	1.134	0.670-1.918	0.639	-	-	-
**AFP**	1.660	1.171-2.354	**0.004***	1.450	1.013-2.076	**0.042***
**Tumor size**	2.073	1.448-2.967	**<0.001***	1.698	1.153-2.500	**0.007***
**No. of tumors**	2.122	1.406-3.202	**<0.001***	1.903	1.241-2.918	**0.003***
**MVI**	2.028	1.407-2.923	**<0.001***	1.775	1.208-2.609	**0.004***
**Edmondson-Steiner classification**	1.657	1.161-2.366	**0.005***	1.796	1.254-2.571	**0.001***
**FCN3 expression**	0.298	0.205-0.432	**<0.001***	0.446	0.294-0.675	**<0.001***

* Significant results ($P < 0.05$) are given in bold.

Abbreviations: HR, hazard risk ratio; CI, confidence interval.

Table S2: Primer sequences used in this study.

Gene	Sequence (5'-3')
GAPDH-F	GTCTCCTCTGACTTCAACAGCG
GAPDH-R	ACCACCCTGTTGCTGTAGCCAA
FCN3-F	GTGAGCCAGGAGATCCAGTGAAC
FCN3-R	GCCCTCAGGTAGGCACAGAT
SBDS-F	CAGTGCGTTTGGAACAGATGACC
SBDS-R	CCTAAACATCTGCTCCAGTTGTG
YBX1-F	GCGAAGGTTCCCACCTTACT
YBX1-R	GTTGTCAGCACCCTCCATCA
EIF6-F	CCCAGACACAGTGCAGATTAG
EIF6-R	AAGACTTCCACCTTGAGCAC
siFCN3#1	GCAUCCUGUUACCGAUCAAAU
	UUGAUCGGUAACAGGAUGCAU
siFCN3#2	AGUCAAUGCCAUAUUUGUGGG
	CACAAAUAUGGCAUUGACUGG
siYBX1#1	GGAGUUUGAUGUUGUUGAAGG
	UUCAACAACAUCAAACUCCAC
siYBX1#2	CAAGGAAGAUGUAUUUGUACA
	UACAAAUACAUCUUCCUUGGU
siControl (Scramble)	UUCUCCGAACGUGUCACGUTT
	ACGUGACACGUUCGGAGAATT
ChIP-Primer-F	TGAGCATTTAGAAGCAAGAGAAGG
ChIP-Primer-R	ATGGAGCAAGCACAACAAAACAA

Table S3: Primary antibodies used in this study.

Antibody	Company	Cat No.	Application
FCN3	Biorbyt	Orb450180	IHC: 1/100
FCN3	Proteintech	11867-1-AP	WB: 1/500
Beta-Tubulin	Proteintech	10094-1-AP	WB: 1/2000
p53	Proteintech	60283-2-Ig	WB: 1/2000
Bax	Proteintech	60267-1-Ig	WB: 1/5000;<br>IHC: 1/500
Bcl2	Proteintech	60178-1-Ig	WB: 1/2000;<br>IHC: 1/10000
Cleaved caspase3	Cell signaling technology	#9664	WB: 1/1000;<br>IHC: 1/2000
His-tag	ABclonal	AE003	WB: 1/2000; IP: 1/50
Myc-tag	ABclonal	AE010	WB: 1/500; IP: 1/50
SBDS	Proteintech	17618-1-AP	WB: 1/500; IP: 1/200
EIF6	ABclonal	A1818	WB: 1/500; IF: 1/50
YBX1	Proteintech	20339-1-AP	WB: 1/500; IP: 1/500
RPL5	Proteintech	29092-1-AP	WB: 1/500
RPL11	Proteintech	16277-1-AP	WB: 1/500; IP: 1/50
RPL23	Proteintech	16086-1-AP	WB: 1/500
MDM2	Proteintech	66511-1-Ig	WB: 1/1000

Fig.S1

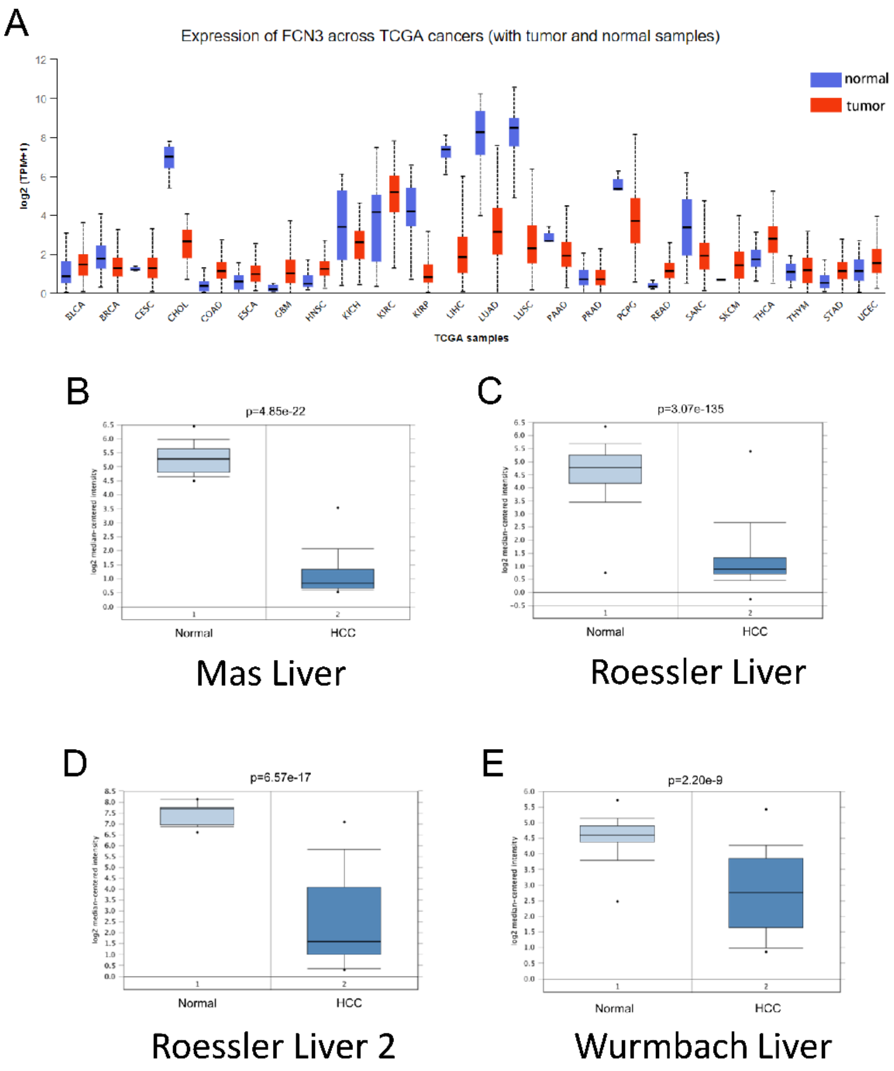
A
Expression of FCN3 across TCGA cancers (with tumor and normal samples)
normal
tumor
log2 (TPM+1)
BLCA BRCA CESC CHOL COAD ESCA GBM HNSC KICH KIRC KIRP LIHC LUAD LUSC PAAD PRAD PCPG READ SARC SKCM THCA THYM STAD UCEC
TCGA samples
B
p=4.85e-22
Normal
HCC
Mas Liver
C
p=3.07e-135
Normal
HCC
Roessler Liver
D
p=6.57e-17
Normal
HCC
Roessler Liver 2
E
p=2.20e-9
Normal
HCC
Wurmbach Liver

Fig.S2

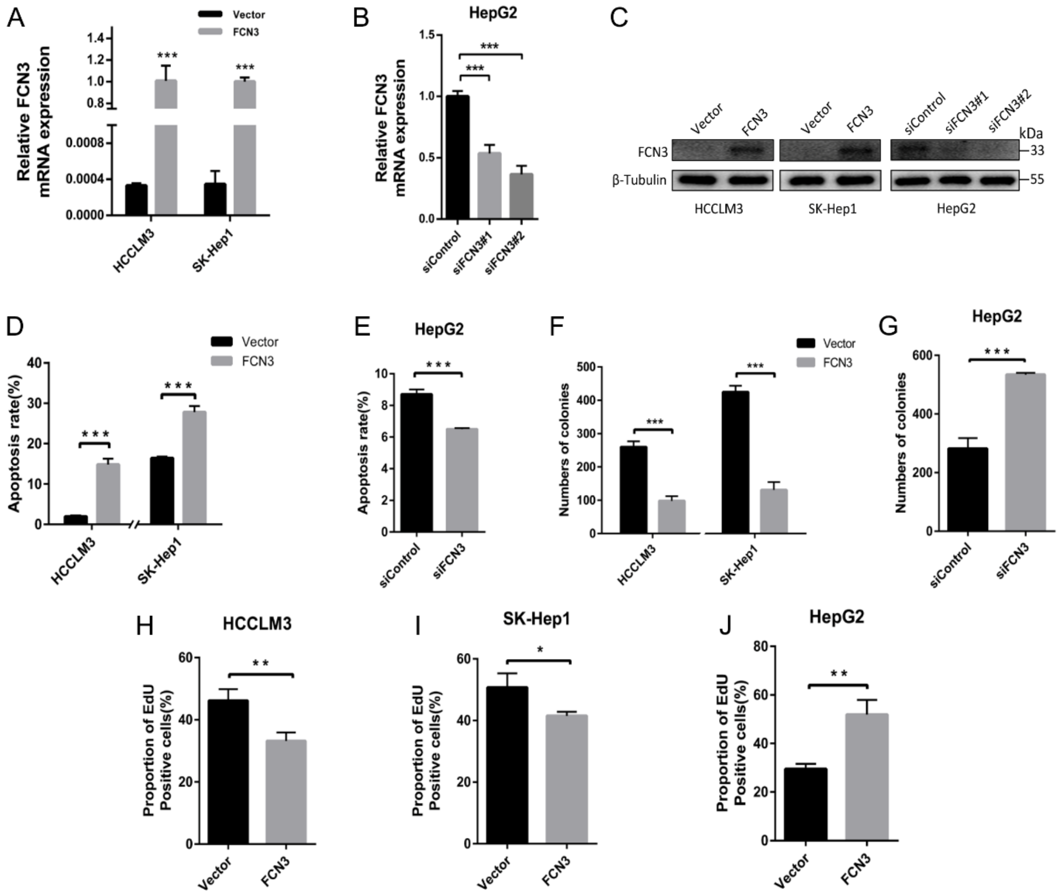

A
Vector
FCN3
Relative FCN3 mRNA expression
HCCLM3
SK-Hep1
B
HepG2
Relative FCN3 mRNA expression
siControl
siFCN3#1
siFCN3#2
C
Vector
FCN3
siControl
siFCN3#1
siFCN3#2
kDa
FCN3
β-Tubulin
33
55
HCCLM3
SK-Hep1
HepG2
D
Apoptosis rate(%)
E
HepG2
Apoptosis rate(%)
siFCN3
F
Numbers of colonies
G
HepG2
Numbers of colonies
H
HCCLM3
Proportion of EdU Positive cells(%)
I
SK-Hep1
J
HepG2

Fig.S3

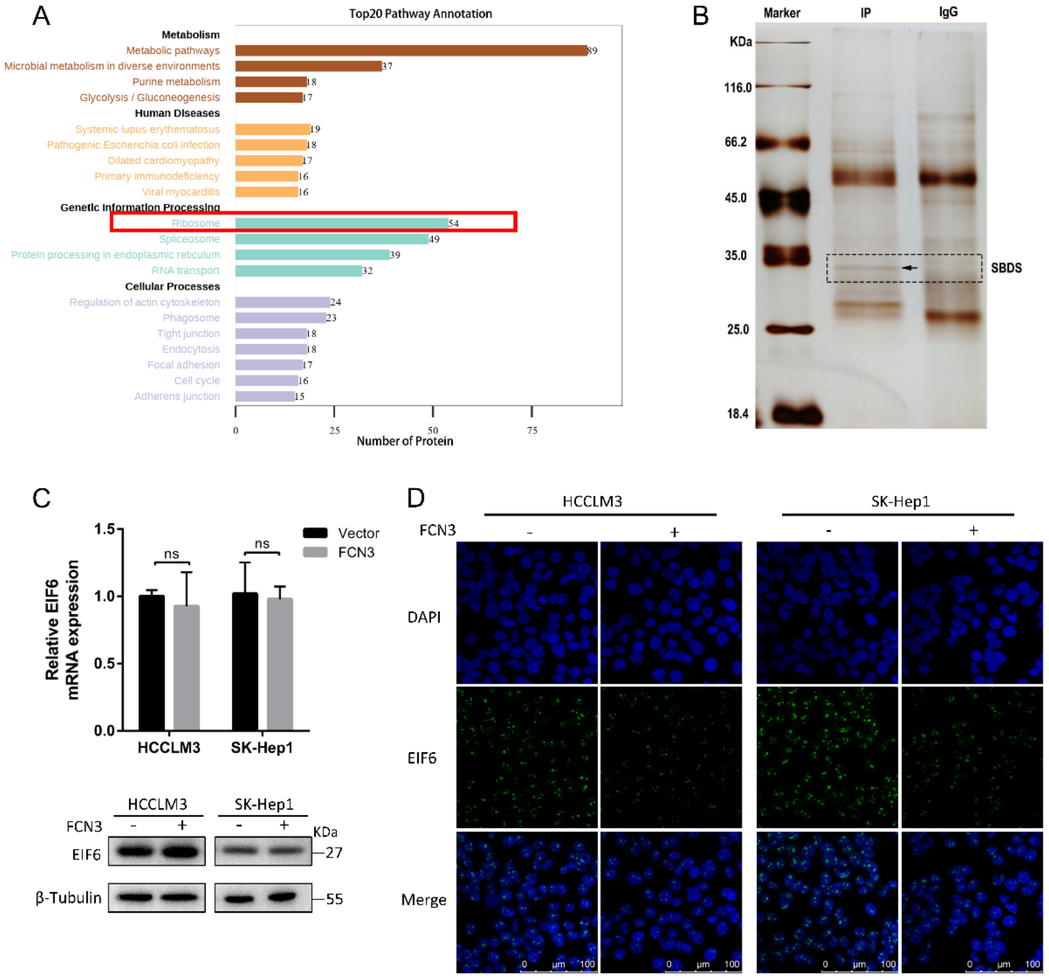

A
Top20 Pathway Annotation
Metabolism
Metabolic pathways
Microbial metabolism in diverse environments
Purine metabolism
Glycolysis / Gluconeogenesis
Human Diseases
Systemic lupus erythematosus
Pathogenic Escherichia coli infection
Dilated cardiomyopathy
Primary immunodeficiency
Viral myocarditis
Genetic Information Processing
Ribosome
Spliceosome
Protein processing in endoplasmic reticulum
RNA transport
Cellular Processes
Regulation of actin cytoskeleton
Phagosome
Tight junction
Endocytosis
Focal adhesion
Cell cycle
Adherens junction
89
37
18
17
19
18
17
16
16
54
49
39
32
24
23
18
18
17
16
15
0
25
50
75
Number of Protein
B
Marker
IP
IgG
KDa
116.0
66.2
45.0
35.0
25.0
18.4
SBDS
C
1.5
1.0
0.5
0.0
Relative EIF6 mRNA expression
ns
ns
Vector
FCN3
HCCLM3
SK-Hep1
HCCLM3
SK-Hep1
FCN3
-
+
-
+
KDa
EIF6
27
β-Tubulin
55
D
HCCLM3
SK-Hep1
FCN3
-
+
-
+
DAPI
EIF6
Merge
0
μm
100

Fig.S4

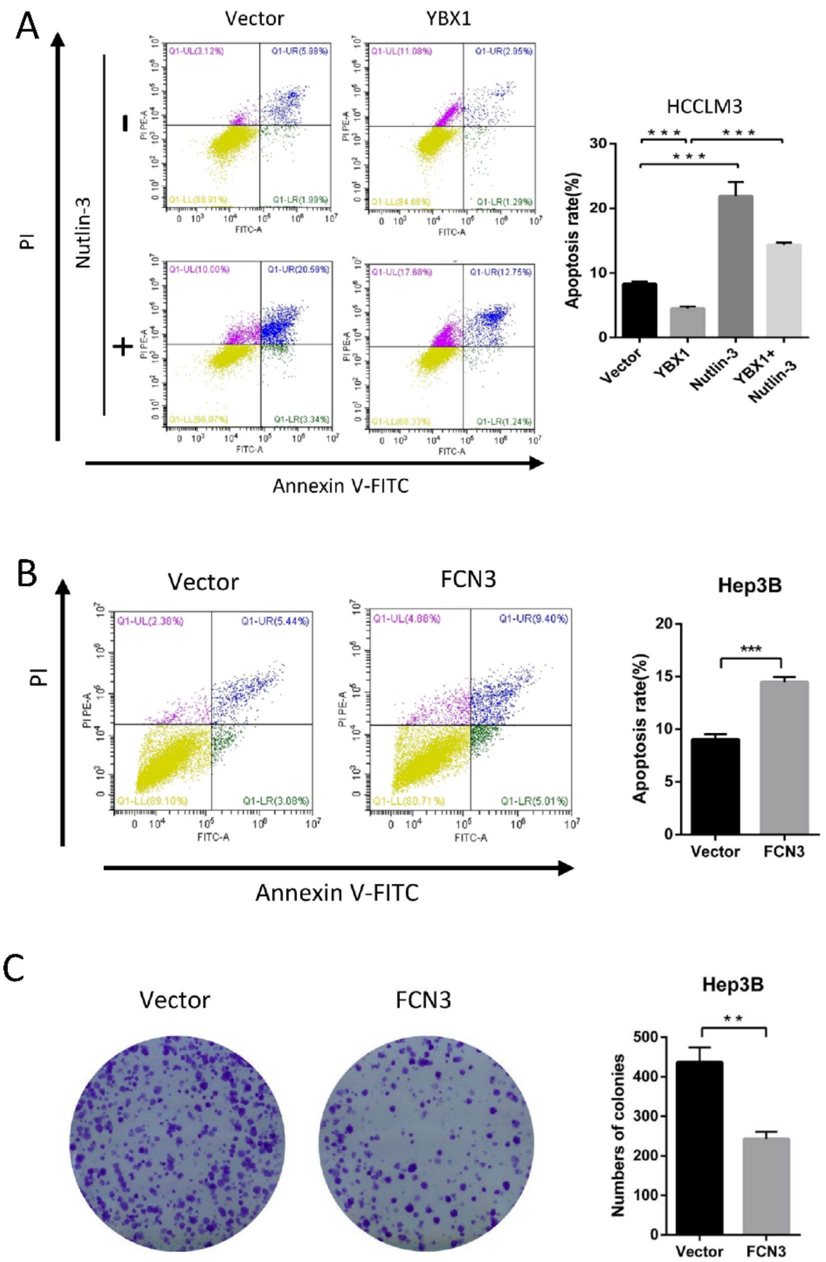
A
Vector
YBX1
PI
Nutlin-3
-
+
Annexin V-FITC
HCCLM3
Apoptosis rate(%)
Vector
YBX1
Nutlin-3
YBX1+ Nutlin-3
B
Vector
FCN3
PI
Annexin V-FITC
Hep3B
Apoptosis rate(%)
Vector
FCN3
C
Vector
FCN3
Hep3B
Numbers of colonies
Vector
FCN3
